# The International Outcome Inventory for Hearing Aids (IOI-HA) and Its Correlation With World Health Organization Quality of Life – Brief Version (WHOQOL-BREF) in Assessing the Quality of Life of Patients With Hearing Loss Using Hearing Aids

**DOI:** 10.7759/cureus.90138

**Published:** 2025-08-15

**Authors:** Ahmad M Alrasheed, Khalid Ardi, Omar Z Alaidaroos, Montasir Junaid, Salmah M Alharbi, Mona Alshehri, Amirah Abumismar, Rakan M Alrasheed

**Affiliations:** 1 Otolaryngology - Head and Neck Surgery, Diriyah Hospital, Riyadh Third Health Cluster, Riyadh, SAU; 2 Otolaryngology - Head and Neck Surgery, King Faisal Medical City For Southern Regions, Abha, SAU; 3 Otolaryngology - Head and Neck Surgery, King Abdulaziz Hospital Jeddah, Jeddah, SAU; 4 Otolaryngology - Head and Neck Surgery, Armed Forces Hospital Southern Region, Khamis Mushait, SAU; 5 Otolaryngology - Head and Neck Surgery, Asir Central Hospital, Abha, SAU; 6 Otolaryngology, Aseeer, Abha, SAU; 7 Family Medicine, Family Medicine Academy-Qassim Health Cluster, Buraidah, SAU; 8 Medicine, Vision Colleges, Riyadh, SAU

**Keywords:** audiological outcomes, hearing aids, hearing loss, ioi-ha, quality of life, saudi arabia, whoqol-bref

## Abstract

Background

The auditory impairment can have a significant impact on communication, mental health, and interpersonal interaction. One of the main interventions to combat these effects is hearing aids, although their real-life efficiency and their impact on the quality of life differ among users. In this study, the efficacy of hearing aids and their correlation with the World Health Organization Quality of Life - Brief version (WHOQOL-BREF) in hearing aid wearers were assessed using the International Outcome Inventory-Hearing Aids (IOI-HA).

Methods

The study was performed as cross-sectional research at the Armed Forces Hospital-Southern Region (AFHSR), Khamis Mushait, Saudi Arabia, between August 2021 and May 2022. A sample of 210 adults of the age group between 18 to 65 years with confirmed hearing loss and prescribed conventional hearing aids was used. The interviews and electronic medical record data were considered, in which demographics, hearing aid features, IOI-HA scores, and WHOQOL-BREF answers were considered. Data were analyzed by SPSS version 22 (IBM Corporation, Armonk, NY).

Results

The average IOI-HA score was 23.8 ± 6.4, with 41.9% (88 participants) of wearers using hearing aids more than eight hours a day and 30.5% (64 participants) claiming the considerable assistance of their aids. Moderate hearing loss, longer hearing aid use, and increased perception of benefit emerged as the factors relating to higher IOI-HA scores. The IOI-HA and WHOQOL-BREF scores showed a positive association, with the psychological and social areas showing the strongest link.

Conclusion

Hearing Aids effectively improve the lives of people with decreased hearing, especially after over a year of usage. Combining hearing-specific outcome measures with measures of the broader quality of life gives a better picture of the patient experience and can drive more personalized and effective strategies of hearing rehabilitation.

## Introduction

Hearing impairment is a common disability affecting human senses and may also reduce the quality of life of a person to a large extent [1]. It influences communication, social interaction, and psychological well-being, especially when they are not addressed [1, 2]. Hearing aids (HAs) are among the most widespread rehabilitative solutions to hearing-impaired people, but they are characterized by efficacy beyond their auditory effects and can be linked to psychosocial and functional enhancements in general [2]. Nonetheless, the process of subjective experience of hearing aid users is different, and it is necessary to consider not only the results in the auditory sphere but also life activities and well-being [3]. One of the standardized and validated tools is the International Outcome Inventory-Hearing Aids (IOI-HA), which was designed to measure the outcomes of using hearing aids in real life in various aspects, including the usage, benefit, satisfaction, residual limitation, and the quality of life [4, 5]. It has found wide use in clinical practice and research due to its simplicity and its universal applicability [6]. The IOI-HA does not fully address other facets of well-being, such as psychological, social, and environmental health, despite offering insightful scientific information about users' experiences with hearing aids [7, 8].

The World Health Organization short form of the multidomain assessment of quality of life, known as WHOQOL-BREF, gauges the quality of life within four domains, which include the physical, psychological, social, and environmental well-being [8, 9]. The tool functions well in supplementing the IOI-HA because it gives a wider picture of what patients experience overall, rather than their outcomes related specifically to hearing [10]. Through examining the relationship between IOI-HA scores and the WHOQOL-BREF domains, researchers will be able to acquire a more holistic picture of the role that hearing aid use plays in all aspects of life, not merely hearing-related outcomes [11]. Therefore, this research would be carried out as a result of seeking to evaluate this connection to achieve a better comprehension of how the use of the hearing aid improves or impacts the life of the subjects participating in the study with hearing loss than restricting the report to focused findings on the nationality group or center-based population. By using this method, we intend to determine whether subjective results from hearing aids are in line with more thorough health-related quality of life data and the degree to which wearing hearing aids is linked to a higher quality of life. Clinicians, audiologists, and public health policymakers may find this finding useful to tailor interventions and follow-up programs towards improving the satisfaction of hearing impaired both on auditory and life satisfaction levels.

## Materials and methods

Study design

This cross-sectional analytical study was carried out at a Saudi tertiary care hospital to assess the results of wearing hearing aids and investigate their relationship with quality of life. The study focused on both the hearing-related and general life consequences to present a complete picture of the real-world consequences of hearing aids. This design enabled analysis of the associations between clinical and demographic variables and hearing aid outcomes at one point in time.

Setting and timeframe of research

The Armed Forces Hospital-Southern Region (AFHSR) in Saudi Arabia's Aseer region served as the study's site. One of the largest tertiary public medical facilities in the province, the AFHSR offers free medical care to citizens, military personnel, and their families. Four million individuals are served by the hospital [12]. Interviews with the patients took place between August 2021 and May 2022.

Population

The study population consisted of adults aged 18-65 who were diagnosed with hearing loss at AFHSR between 2017 and 2019 and who were prescribed hearing aids by an otolaryngologist. Of the 361 eligible individuals contacted, 210 gave their consent to take part in the study, resulting in a 58.1% response rate. Participants have a prescription for hearing aids and a documented hearing threshold based on pure-tone audiometry to meet the inclusion criteria. Individuals under the age of 18 or older than 65, those ineligible for AFHSR services, those with implanted hearing devices, patients without pre-fitting audiometric records, and those prescribed hearing aids just for tinnitus masking were among the exclusion criteria.

Data variables and collection procedures

Age, sex, educational attainment, co-morbidities, the type and severity of hearing loss, and the features of hearing aids (type, duration of use, side used) were among the clinical and demographic information that was taken from electronic health records. Specific hearing aid outcomes, such as usage duration, perceived benefit, satisfaction, impact on activity limitations, and social engagement, were evaluated using the IOI-HA [13] (Appendix). The WHOQOL-BREF [14] evaluated general quality of life in four areas. Depending on the participants' accessibility, interviews were conducted in-person or online and lasted roughly thirty minutes. Confidentiality was always maintained, and informed consent was acquired either orally or through chat.

Data analysis

IBM SPSS version 22 (IBM Corporation, Armonk, NY), a statistical tool, was used to modify, code, and input the data once it had been extracted. In the statistical analysis, two-tailed tests were employed. A statistically significant P value was defined as less than 0.05. Descriptive analysis based on frequency and percent distribution was performed on the participants' biodemographic data, hearing loss data, first aid use, clinical symptoms, and IOI-HA items. Each person's overall IOI-HA score (out of 35) was displayed along with its range, mean, and standard deviation. For variables with more than two categories, One-Way Analysis of Variance (ANOVA) was utilized to test for statistically significant differences between groups; for comparisons between two groups, the independent t-test was employed. A P value of less than 0.05 was deemed statistically significant for all two-tailed statistical tests.

## Results

The total number of participants was 210. With a mean age of 48.8 ± 13.5 years, the participants' ages varied from 18 to 65. Specifically, 183 individuals (87.1%) were married, while 115 participants (54.8%) were men. Twenty-one (10%) had a university degree, whereas 126 (60%) had less than a high school diploma. There were 112 (53.3%) people without any chronic health issues, 52 (24.8%) with hypertension, 12 (5.7%) with hypothyroidism, and 65 (31%) with diabetes (Table 1).

**Table 1:** Participants' Bio-demographic Data

Bio-demographic Data	No	%
Age in Years		
< 40	50	23.8%
40-59	98	46.7%
60+	62	29.5%
Gender		
Male	115	54.8%
Female	95	45.2%
Marital Status		
Married	183	87.1%
Not Married	27	12.9%
Educational Level		
Below High School	126	60.0%
Diploma/High School	63	30.0%
University /Above	21	10.0%
Chronic Diseases		
None	112	53.3%
Diabetes Mellitus	65	31.0%
Hypertension	52	24.8%
Hypothyroidism	12	5.7%
Renal Disease	4	1.9%
Asthma	1	.5%
Depression	5	2.4%
Heart Disease	2	1.0%
Others	7	3.3%

Of the research participants, 57 individuals (27.1%) had severe hearing loss, and 72 individuals (34.3%) had moderate hearing loss on the right side. In 140 participants (72.2%) of instances affecting the right side, it was sensorineural hearing loss (SNHL). On the left side, 52 participants (24.8%) of the patients had severe hearing loss, or SNHL, in 145 cases (73.6%) of the instances, while 76 participants (36.2%) had moderate hearing loss. Forty-two (20%) of the subjects had bilateral hearing loss. In the ear (ITE) hearing aids were prescribed to 43 participants (20.5%), and behind-the-ear (BTE) were prescribed to 159 participants (75.7%). Diagnosis for which the hearing aids were prescribed was known to 163 (77.6%) of study participants, and 135 of our participants have used their hearing aids regularly for more than a year. Table 2 displays the characteristics of hearing loss and the use of hearing aids by Saudi Arabian study participants.

**Table 2:** Participants' Use of Hearing Aids and Hearing Loss CHL: Conductive Hearing Loss, SNHL: Sensorineural Hearing Loss

Hearing Loss and Hearing Aids Data		No	%
Right Ear Hearing Loss Degree	Normal	16	7.60%
	Mild	27	12.90%
	Moderate	72	34.30%
	Moderately severe	16	7.60%
	Severe	57	27.10%
	Profound	22	10.50%
Right Ear Hearing Loss Type	CHL	17	8.80%
	SNHL	140	72.20%
	Mixed	37	19.10%
Left Ear Hearing Loss Degree	Normal	13	6.20%
	Mild	27	12.90%
	Moderate	76	36.20%
	Moderately severe	16	7.60%
	Severe	52	24.80%
	Profound	26	12.40%
Left Ear Hearing Loss Type	CHL	16	8.10%
	SNHL	145	73.60%
	Mixed	36	18.30%
Side of Prescribed Hearing Aid	Right	93	44.30%
	Left	75	35.70%
	Bilateral	42	20.00%
Type of Hearing Aid	Behind The Ear	159	75.70%
	In The Ear	42	20.50%
	In The Canal	6	2.90%
	Completely In Canal	2	1.00%
Duration of Hearing Aid Use	< 1 month	6	2.90%
	3 months	13	6.20%
	6 months	23	11.00%
	1 year	33	15.70%
	> 1 year	135	64.30%
Patient's Awareness of The Diagnosis for Which Hearing Aids Were Prescribed	Yes	163	77.60%
	No	10	4.80%
	Not sure	37	17.60%

Twenty-four patients (11.4%) had auditory trauma, while 17 cases (8.1%) had head trauma. New worsening of hearing (40%), tinnitus (28.6%), vertigo/dizziness (21%), wax impaction while wearing HA (17.6%), and pus ear discharge (11%) were the most common symptoms reported within the previous two weeks before the interview. A total of 27 people (12.9%) had ear surgery. Regarding medical consultations, 76 (36.2%) attend the ENT/audiology clinic every year, whilst 88 (41.9%) never do so. Table 3 shows the clinical symptoms and risk factors for hearing aid users who have received clinical consultation.

**Table 3:** Risk Factors, Clinical Symptoms Among Hearing Aid Users ENT: Ear, Nose, Throat

	Count	Column N %
Exposure to		
Acoustic Trauma	24	11.4%
Head Trauma	17	8.1%
Both of Them	5	2.4%
None	164	78.1%
Symptoms in the Past 2 Weeks		
None	52	24.8%
New Worsening of Hearing	84	40.0%
Tinnitus	60	28.6%
Vertigo/Dizziness	44	21.0%
Wax Impaction While Wearing Hearing Aids	37	17.6%
Pus Ear Discharge	23	11.0%
Alternative treatment was offered		
Yes	62	29.5%
No	148	70.5%
Type of Alternative Treatment		
Non-Surgical	33	53.2%
Surgical	29	46.8%
Undergone Ear Surgeries		
Yes	27	12.9%
No	183	87.1%
Frequency of ENT/Audiology Clinic Visit		
Never	88	41.9%
Every month	3	1.4%
Every 3 months	7	3.3%
Every 6 months	36	17.1%
Every year	76	36.2%

Approximately 88 participants (41.9%) of research participants reported using HA for more than eight hours per day, 64 participants (30.5%) reported it was very helpful, 39 participants (18.6%) reported no issues, and 25 participants (11.9%) believed it was worth the trouble. However, 25 participants (11.9%) of users said that their ability to accomplish some tasks was unaffected by their hearing impairments, 90 participants (42.9%) said that their hearing was not a hindrance, and 46 participants (21.9%) said that wearing HA significantly improved their quality of life. Out of 35, the average score was 23.8 ± 6.4 (68%). Table 4 shows the summary of participants' answers to the IOI-HA questionnaire.

**Table 4:** International Outcome Inventory-Hearing Aid (IOI-HA) Score Among Hearing Aid Users SD: Standard Deviation

IOI-HA Factors		No	%
On an average day, how many hours did you use the hearing aid(s)?	< 1 hour / day	52	24.8%
	1-4 hours / day	40	19.0%
	4-8 hours / day	30	14.3%
	> 8 hours / day	88	41.9%
Over the past two weeks, how much has the hearing aid helped?	Helped not at all	39	18.6%
	Helped slightly	48	22.9%
	Helped Moderately	42	20.0%
	Helped Quite A Lot	17	8.1%
	Helped Very Much	64	30.5%
When you use your present hearing aid(s), how much difficulty do you still have in that situation?	Very Much Difficulty	37	17.6%
	Quite A Lot of Difficulty	30	14.3%
	Moderate Difficulty	59	28.1%
	Slight Difficulty	45	21.4%
	No Difficulty	39	18.6%
Do you think your present hearing aid(s) is worth the trouble?	Not At All Worth It	35	16.7%
	Slightly Worth It	34	16.2%
	Moderately Worth It	46	21.9%
	Quite A Lot Worth It	70	33.3%
	Very Much Worth It	25	11.9%
How much have your hearing difficulties affected the things you can do?	Affected Very Much	30	14.3%
	Affected Quite A Lot	22	10.5%
	Affected Moderately	46	21.9%
	Affected Slightly	59	28.1%
	Affected Not At All	53	25.2%
How much do you think other people were bothered by your hearing difficulties?	Bothered Very Much	32	15.2%
	Bothered Quite A Lot	20	9.5%
	Bothered moderately	19	9.0%
	Bothered Slightly	49	23.3%
	Bothered Not At All	90	42.9%
How much has your present hearing aid(s) changed your enjoyment of life?	Worse	0	0.0%
	No Change	33	15.7%
	Slightly Better	46	21.9%
	Quite A Lot Better	85	40.5%
	Very Much Better	46	21.9%
Overall score	Range	9-35	
	Mean ± SD	23.8 ± 6.4	

Those with moderately severe right ear hearing loss had significantly higher mean IOI-HA scores (26.7 ± 4.7; P = 0.049), according to the research, which used One-Way ANOVA and independent t-tests. Furthermore, the mean scores of individuals who had been wearing hearing aids for more than a year were significantly higher (25.9 ± 5.4; P = 0.001). Additionally, those who were unsure of why they were wearing their hearing aids also had noticeably higher mean scores (25.7 ± 5.0; P = 0.023). Factors related to research participants' IOI-HA scores are shown in Table 5.

**Table 5:** Factors Associated With International Outcome Inventory-Hearing Aid (IOI-HA) Score Among Study Participants IOI-HA: International Outcome Inventory-Hearing Aids, CHL: Conductive Hearing Loss, SNHL: Sensorineural Hearing Loss

Factors		IOI-HA Score		p-value
		Mean	SD	
Age in years	< 40	24.3	6.3	0.344
	40-59	23.1	6.7	
	60+	24.4	6.0	
Gender	Male	23.6	6.5	0.640
	Female	24.0	6.3	
Marital status	Married	23.8	6.4	0.883
	Not married	23.6	6.3	
Educational level	Below high school	24.4	6.2	0.165
	Diploma/ high school	22.8	6.4	
	University / above	22.6	7.1	
Right Ear Hearing Loss Degree	Normal	21.2	7.3	0.049
	Mild	25.4	5.0	
	Moderate	23.0	6.1	
	Moderately severe	26.7	4.7	
	Severe	24.2	6.5	
	Profound	22.9	8.0	
Right Ear Hearing Loss Type	CHL	24.2	5.7	0.498
	SNHL	23.7	6.4	
	Mixed	25.0	6.3	
Left Ear Hearing Loss Degree	Normal	24.5	7.4	0.548
	Mild	25.7	5.3	
	Moderate	22.9	5.9	
	Moderately severe	23.6	7.1	
	Severe	23.9	6.6	
	Profound	23.7	7.4	
Left Ear Hearing Loss Type	CHL	24.0	5.3	0.718
	SNHL	23.5	6.3	
	Mixed	24.4	6.9	
Side of Used Hearing Aid	Right	24.0	6.3	0.191
	Left	22.8	7.0	
	Bilateral	25.0	5.2	
Duration of Hearing Aid Use	< 1 month	16.5	6.0	0.001
	3 months	15.5	5.1	
	6 months	19.5	5.7	
	1 year	22.7	5.9	
	> 1 year	25.9	5.4	
Patient's Awareness of The Diagnosis for Which Hearing Aids Were Prescribed	Yes	24.2	6.4	0.023
	No	25.7	5.0	
	Not sure	21.2	6.2	

## Discussion

In this research study, the WHOQOL-BREF and IOI-HA were administered to assess the correlation between quality of life and the hearing aids effectiveness. The overall IOI-HA score was analyzed, and it was found that the average of the score is 23.8 ± 6.4, which shows positive satisfaction levels about the use of the hearing aids among the participants of the study. This observation is in line with a population-based study of Houmøller et al. [15], who also validated the IOI-HA and found similar outcomes scores between groups. Furthermore, the majority of hearing aid users reported satisfactory results and daily usage exceeding eight hours, according to earlier research by Houmøller et al. [16]. This pattern was mirrored in our study, where 41.9% (88 participants) reported wearing their hearing aids for more than eight hours daily. The constancy in usage patterns emphasizes the significance of daily hearing aid use in improving patient-reported outcomes, even though Houmøller et al.'s study focused predominantly on older persons with presbycusis and did not report population-specific IOI-HA scores. 

According to our survey, 64 (30.5%) of participants said their hearing aids were very helpful to them, and 46 participants (21.9%) said they made their lives much better by using them. This result corresponds with those of Brannstrom et al. [17] and Pronk et al. [18], who have found that regular use of hearing aids results in a significant increase in the ability to communicate and enjoy life. Interestingly, those who had been using their hearing aids for over a year had considerably higher scores on the IOI-HA, which again confirms that adaptation and long-term usage are important, and so it was in the research of Dornhoffer et al. [19], who observed higher results with longer use duration and better fitting support.

Interestingly, the results suggest that people with moderately severe hearing loss reported the best average IOI-HA scores, which implies that they experienced better perceived outcomes compared to people with milder or more severe hearing loss. It is consistent with the results prior study by Barnett et al. [20], showing that hearing aid satisfaction remains low when compared with the degree of hearing loss, where little to no amplification is useful in profound impairment. These results accentuate the significance of meticulously aligning hearing aid services to the level of loss and identifying alternative interventions to use in individuals with a severe to profound loss.

In the overall quality of life, WHOQOL-BREF scores were positively correlated with higher scores of IOI-HA, including the psychological and social areas. This validates a study by Vas et al. [21], which revealed that users of hearing aids usually experience not only improved interpersonal communication but also in social interaction and mood. Our findings support the significance of the combination of audiological treatment and the use of psychosocial support to achieve better general results. In addition, the results demonstrated that patients who routinely visited the ENT/audiology department reported improved performance from their hearing aids. This is in line with the findings of Barker et al. [22], who highlighted the significance of continuous counseling and maintenance.

Finally, 64.3% (135 participants) of the subjects of study had used their hearing aids for more than one year, and thus, the findings of the study did not naturally evaluate the influence of educational level and chronic co-morbidities on hearing aid performance or the level of learning of participants. Future investigations might, thus, play a role in evaluating the effects of health literacy and co-morbidities on compliance with wearing hearing aids and patient satisfaction, particularly in groups with different educational levels and health conditions [23]. Future studies are also advised to use longitudinal studies to monitor the changes in hearing aid outcomes and quality of life over time, since cross-sectional studies are not able to freely conclude as to causation. Also, investigating the results of interventions among individuals with severe to profound hearing impairments, where the effectiveness of standard hearing aids may be too limited, would be useful to determine the compatible interventions and enhance overall patient care plans. The main strengths of this study are the combination in the design of two validated assessments, IOI-HA and WHOQOL-BREF, which help to determine hearing aid device-specific outcomes and broad quality-of-life categories measured in a clinical subject in the territory of an actual health care establishment. The relatively wide sample size of the study and the varied patient profile of a major tertiary care facility also contribute to the greater reliability and generalizability of the results. The cross-sectional design restricts causative inferences, and the use of self-reported measures creates the risk of recall or reporting bias. Future research is challenged to utilize qualitative approaches in addition to the quantification tools to better understand patients' lived experiences, satisfaction, and impediments to productive hearing aid use.

## Conclusions

As evidenced by the IOI-HA scores and positive correlations with WHOQOL-BREF domains, especially in the areas of psychological and social well-being, this study showed that daily use of hearing aids improves the quality of life for people with hearing loss. The participants with longer duration of use of hearing aids and with moderate hearing loss were more satisfied and found more benefit. These results indicate the efficiency of traditional hearing aids in a real-life context and underline the necessity of further support, follow-up, and patient education to maximize the results. The combination of audiological and quality-of-life measurements used in clinical work could contribute to the adaptation of the rehabilitation approach and the enhancement of patient satisfaction.

## Figures and Tables

**Figure 1:**
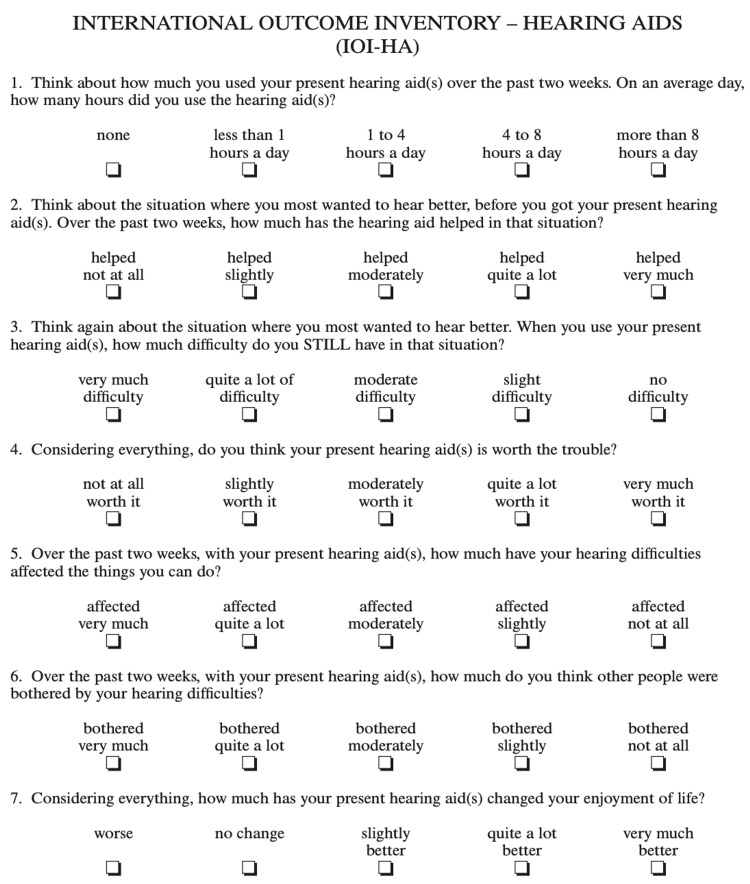
International Outcome Inventory-Hearing Aids (IOI-HA) Questionnaire [13].
